# Evaluation of Seasonal Respiratory Virus Activity Before and After the Statewide COVID-19 Shelter-in-Place Order in Northern California

**DOI:** 10.1001/jamanetworkopen.2020.35281

**Published:** 2021-01-25

**Authors:** Elizabeth Partridge, Ellen McCleery, Ritu Cheema, Natasha Nakra, Satyan Lakshminrusimha, Daniel J. Tancredi, Dean A. Blumberg

**Affiliations:** 1 Department of Pediatrics, University of California at Davis School of Medicine, Sacramento

## Abstract

**Importance:**

Public health initiatives that include shelter-in-place orders are expensive and unpopular. Demonstrating the success of these initiatives is essential to justify their systemic or individual cost.

**Objective:**

To examine the association of a shelter-in-place order with lower rates of seasonal respiratory viral activity.

**Design, Setting, and Participants:**

This cohort study with interrupted time series analysis obtained monthly counts of respiratory virus testing results at UC Davis Health from August 1, 2014, to July 31, 2020. Patients of all ages underwent testing conducted by the laboratory at UC Davis Health, a referral center for a 65 000-square-mile area that includes 33 counties and more than 6 million Northern California residents.

**Exposures:**

A statewide shelter-in-place order was instituted on March 19, 2020, restricting residents to their homes except for traveling for essential activities. Large social gatherings were prohibited, schools were closed, and nonessential personnel worked remotely. Those who had to leave their homes were mandated to wear face masks, engage in frequent handwashing, and maintain physical distancing.

**Main Outcomes and Measures:**

Positivity rates of common respiratory viruses within the community served by UC Davis Health.

**Results:**

A total of 46 128 tests for viral respiratory pathogens over a 6-year period were included in the analysis. For the postexposure period (March 25-July 31), approximately 168 positive test results occurred for the studied organisms in the 2020 virus year, a positivity rate of 9.88 positive results per 100 tests that was much lower than the positivity rate of 29.90 positive results per 100 tests observed for this date range in the previous 5 virus years. In contrast, the positivity rates were similar for the preexposure time frame (August 1-March 24) in the 2020 virus year and for the same time periods in the 5 previous years (30.40 vs 33.68 positive results per 100 tests). In the regression analyses, statistically significant decreases in viral activity were observed in the postexposure period for influenza (93% decrease; incidence rate ratio [IRR], 0.07; 95% CI, 0.02-0.33) and for rhinovirus or enterovirus (81% decrease; IRR, 0.19; 95% CI, 0.09-0.39) infections. Lower rates of postexposure viral activity were seen for respiratory syncytial virus, parainfluenzavirus, coronaviruses, and adenoviruses; however, these associations were not statistically significant.

**Conclusions and Relevance:**

Using interrupted time series analysis of testing for viral respiratory pathogens, this study found that statistically significant lower rates of common community respiratory viruses appeared to be associated with a shelter-in-place order during the coronavirus disease 2019 pandemic.

## Key Points

**Question:**

Was the severe acute respiratory syndrome coronavirus 2 (SARS-CoV-2)–related shelter-in-place order associated with lower rates of common respiratory viruses in a community?

**Findings:**

In this cohort study using interrupted time analysis of 46 128 tests for viral respiratory pathogens, statistically significant lower rates of influenza and rhinovirus or enterovirus infections were found after the institution of a shelter-in-place order to prevent SARS-CoV-2 transmission.

**Meaning:**

Results of this study showed that public health initiatives, such as shelter-in-place orders during a pandemic, appeared to be associated with lower transmission rates of other respiratory viruses in a community.

## Teaser

This cohort study analyzes the implications of pandemic-related stay-at-home restrictions for the positivity rates of influenza and other seasonal respiratory viruses in Northern California.

## Introduction

The 2019-2020 respiratory virus season was like no other in human history, with the emergence of severe acute respiratory syndrome coronavirus 2 (SARS-CoV-2) and its circulation alongside common respiratory viruses. In response to the coronavirus disease 2019 (COVID-19) pandemic, federal, state, and local governments placed restrictions on travel and instituted physical distancing protocols to curb the spread of this novel virus. On March 19, 2020, California governor Gavin Newsom issued a statewide shelter-in-place order.^1^ Under this order, all residents were to remain at home, only leaving to engage in essential activities. As part of this directive, nonessential personnel worked remotely, large social gatherings and worship services were canceled, and California schools were closed. Essential workers and consumers leaving their homes were mandated to wear face masks, engage in frequent handwashing, and maintain a 6-foot distance from people outside their household.^1^ The March 19 stay-at-home order remained in effect through the summer and fall of 2020, with travel permissible only for health care needs; food procurement; outdoor exercise or recreation; outdoor museum visits; and activities at open, low-risk businesses and workplaces for which telework was not possible, including retail, manufacturing, and offices.

The end of the 2019-2020 respiratory virus season presented a unique opportunity to evaluate the outcome of public health interventions in response to SARS-CoV-2 for the epidemiologic characteristics of other circulating respiratory viruses. In this cohort study, we examined the association of the shelter-in-place order with lower rates of seasonal respiratory viral activity using the incidence of common respiratory viruses in a Northern California health system during the statewide shelter-in-place order. Public health initiatives that include shelter-in-place orders are expensive and unpopular. Demonstrating their success is essential to justify their systemic or individual cost.

## Methods

All data used in this study were deidentified, so the study did not qualify as human research according to the policies of the US Department of Health and Human Services or the US Food and Drug Administration; therefore, institutional review board approval was not required. We followed the Transparent Reporting of Evaluations With Nonrandomized Designs (TREND) reporting guideline.

In this cohort study with interrupted time series analysis, we compiled monthly counts of test results for viral respiratory infections conducted by the laboratory at UC Davis Health from August 1, 2014, to July 31, 2020. Patients of all ages underwent this testing. UC Davis Health is a referral center for a 65 000-square-mile area that includes 33 counties and more than 6 million residents. We currently use a respiratory pathogen panel (GenMark ePlex Respiratory Pathogen Panel; GenMark Diagnostics Inc), which tests for influenza A subtypes H1, H1 2019, and H3; influenza B; respiratory syncytial virus (RSV) A and B; parainfluenzavirus 1 to 4; human coronaviruses 229E, HKU1, NL63, and OC43; human metapneumovirus; rhinovirus or enterovirus; and adenovirus. Respiratory syncytial virus and coronaviruses were added to UC Davis Health’s current respiratory pathogen panel in July 2016, limiting our evaluation of these 2 viruses to 5 rather than 7 years. Human metapneumovirus rates during the study period were substantially lower than the rates of other viruses; thus, this organism was not included in the analysis.

Results of point-of-care real-time polymerase chain reaction test for influenza A and B and RSV (cobas Liat PCR System; Roche Diagnostics) were also included in this study. Point-of-care RSV testing was available at UC Davis Health starting in 2018. Respiratory pathogen panel and point-of-care testing data were extracted from clinical encounters in multiple settings, including the hospital, emergency department, and more than 40 outpatient clinics.

### Statistical Analysis

Our interest was in ascertaining whether the seasonally adjusted incidence rate of respiratory virus detection during the shelter-in-place period differed from the expected rates based on previous years. The units of analysis were monthly counts of positive and negative test results. In this study, the shelter-in-place order divided the current virus season into preexposure (August 1, 2019-March 24, 2020) and postexposure (March 25, 2020-July 31, 2020) periods. This calendar time division was used for the 5 previous virus years to permit the comparison of similar calendar periods between virus years while accounting for between-year variation. In the unadjusted analysis of virus incidence, we proportionally allocated the March data in a 24:7 ratio to the preexposure and postexposure periods.

For analyses adjusting for between-year and seasonal effects, we used the GLIMMIX procedure in SAS/STAT software^2^ (SAS Institute Inc) to fit marginal regression models for overdispersed Poisson data with autocorrelated errors for each organism’s monthly time series of positive test result counts. This model included a variable coded 0 for all months before or 1 for all months after March 2020, and coded with the value 7/31 for March 2020, to adjust for the shelter-in-place order affecting the last week of March and fully affecting all weeks subsequent to that. The estimated regression coefficient for this variable was used to estimate the seasonally adjusted incidence rate ratio (IRR) associated with the shelter-in-place order, which was also expressed as a percentage reduction (100 × [1 – IRR]). The Wald test for this regression coefficient was evaluated for statistical significance with a 2-sided α = 5%. All models included additional covariates to adjust for year-to-year differences and cosinor analysis terms to account for sinusoidal seasonal effects.^3^ For each organism, we fit and compared 6 models that varied in how they controlled for historical patterns (categorical year effects vs a linear or quadratic polynomial in calendar time) and how they controlled for seasonal effects (only 12-month cosinor analysis terms or augmented with 6-month terms),^4^ and we used the Akaike information criterion to select the best model.^5^

Offset terms based on the monthly number of definitive test results were included in the model so that that the incidence rates refer to incidence proportions. The small number of indeterminate test results (<0.14%) were excluded from the analysis. In sensitivity analyses, we changed the offset terms to refer to number of days in the month so that incidence rates refer to number of positive test results per day (eTable in the Supplement).

## Results

A total of 46 128 tests for viral respiratory infections over a 6-year period were included in the analysis. For the postexposure period (March 25-July 31), approximately 168 positive test results occurred in the 2020 virus year, a positivity rate of 9.88 positive results per 100 tests, which was much lower than the positivity rate of 29.90 positive results per 100 tests observed for this date range in the previous 5 virus years. In contrast, the positivity rates were similar at the preexposure time frame (August 1-March 24) for the 2020 virus year and for the previous 5 virus years (30.40 vs 33.68 positive results per 100 tests) (Table 1).

**Table 1.** Viral Positivity Rates During Preexposure and Postexposure Periods, Comparison of 2015-2019 vs 2020 Virus Years^a^

Organism	Virus year	Preexposure (August 1-March 24)				Postexposure (March 25-July 31)			
		No. of positive results	No. of tests	No. of positive results/100 tests	No. of positive results/100 d	No. of positive results	No. of tests	No. of positive results/100 tests	No. of positive results/100 d
Influenza	2015-2019	3598.4	25 197.0	14.28	304.69	695.6	8616.0	8.07	95.29
	2020	1762.5	9170.0	19.22	743.67	68.5	1608.0	4.26	46.92
Rhinovirus/enterovirus	2015-2019	3005.0	13 206.7	22.75	254.45	1303.0	5554.3	23.46	178.49
	2020	600.5	3262.6	18.41	253.38	51.5	1095.4	4.70	35.27
RSV	2017-2019	947.7	9076.5	10.44	80.25	114.3	3962.5	2.88	15.66
	2020	363.3	3998.4	9.09	153.29	27.7	1188.6	2.33	18.97
Parainfluenzavirus	2015-2019	559.4	13 288.0	4.21	47.37	281.6	5578.0	5.05	38.58
	2020	109.3	3262.6	3.35	46.12	3.7	1095.4	0.34	2.53
Coronavirus	2017-2019	367.7	8506.2	4.32	31.13	70.3	3822.8	1.84	9.63
	2020	116.7	3262.6	3.58	49.24	7.3	1095.4	0.67	5.00
Adenovirus	2015-2019	199.4	13 283.0	1.50	16.88	153.6	5578.0	2.75	21.04
	2020	58.8	3262.6	1.80	24.81	9.2	1095.4	0.84	6.30
All organisms	2015-2019	8677.7	25 765.3	33.68	734.78	2618.3	8755.7	29.90	358.67
	2020	3011.0	9905.8	30.40	1270.46	168.0	1701.2	9.88	115.07

Abbreviation: RSV, respiratory syncytial virus.

^a^ Virus year begins August 1 of the previous nominal year. For example, virus year 2020 runs from August 1, 2019, through July 31, 2020. This table was based on monthly count data, with March data proportionally allocated 24:7 to the 2 seasons depicted here. The RSV and coronavirus testing data available to us begin with September 2016 (virus year 2017). For other organisms, the time series begins August 2014 (virus year 2015).

We observed lower rates of all incident viral respiratory infections after the institution of the statewide shelter-in-place order compared with the previous 5 to 7 years (2014-2019). Specifically, a statistically significant decrease of 93% (IRR, 0.07; 95% CI, 0.02-0.33) was found in 2020 for laboratory-confirmed cases of influenza and 81% (IRR, 0.19; 95% CI, 0.09-0.39) for laboratory-confirmed cases of rhinovirus or enterovirus. Lower rates of viral activity ranging from 63% to 91% (IRR range, 0.37-0.09) were also observed in all other viral pathogens, but these rates were not statistically significant because of smaller incidence rates, higher seasonal variability, and thus wider CIs (Table 2 and Figure).

**Table 2.** Seasonally Adjusted Data After Institution of Shelter-in-Place Order

Organism	IRR (95% CI)^a^	*P* value	% Reduction in positive cases from expected
Influenza	0.07 (0.02 to 0.33)	.001	93 (67 to 98)
Rhinovirus/enterovirus	0.19 (0.09 to 0.39)	<.001	81 (61 to 91)
RSV	0.33 (0.10 to 1.10)	.07	67 (−10 to 90)
Parainfluenzavirus	0.09 (0.01 to 1.25)	.07	91 (−25 to 99)
Coronavirus	0.37 (0.06 to 2.52)	.30	63 (−152 to 94)
Adenovirus	0.23 (0.04 to 1.23)	.08	77 (−23 to 96)

Abbreviations: IRR, incidence rate ratio; RSV, respiratory syncytial virus.

^a^ For each organism, the IRR was estimated in a Poisson regression model for overdispersed, autocorrelated monthly time series data, with cosinor analysis adjustments for sinusoidal seasonal effects (12-month cycle) and additional adjustment for historical patterns. For rhinovirus or enterovirus, adjustments for historical patterns were made, with calendar date as a linear term, and additional cosinor analysis terms for 6-month cycles were added. For all other organisms, indicator terms for each virus year (August-July) were adjusted for historical patterns, with 2019-2020 as the reference year. For parainfluenzavirus, cosinor analysis terms included those for 6-month cycles. Percentage decreases in the incidence rate of positive cases refers to the number of positive results per number of tests.

**Figure.**
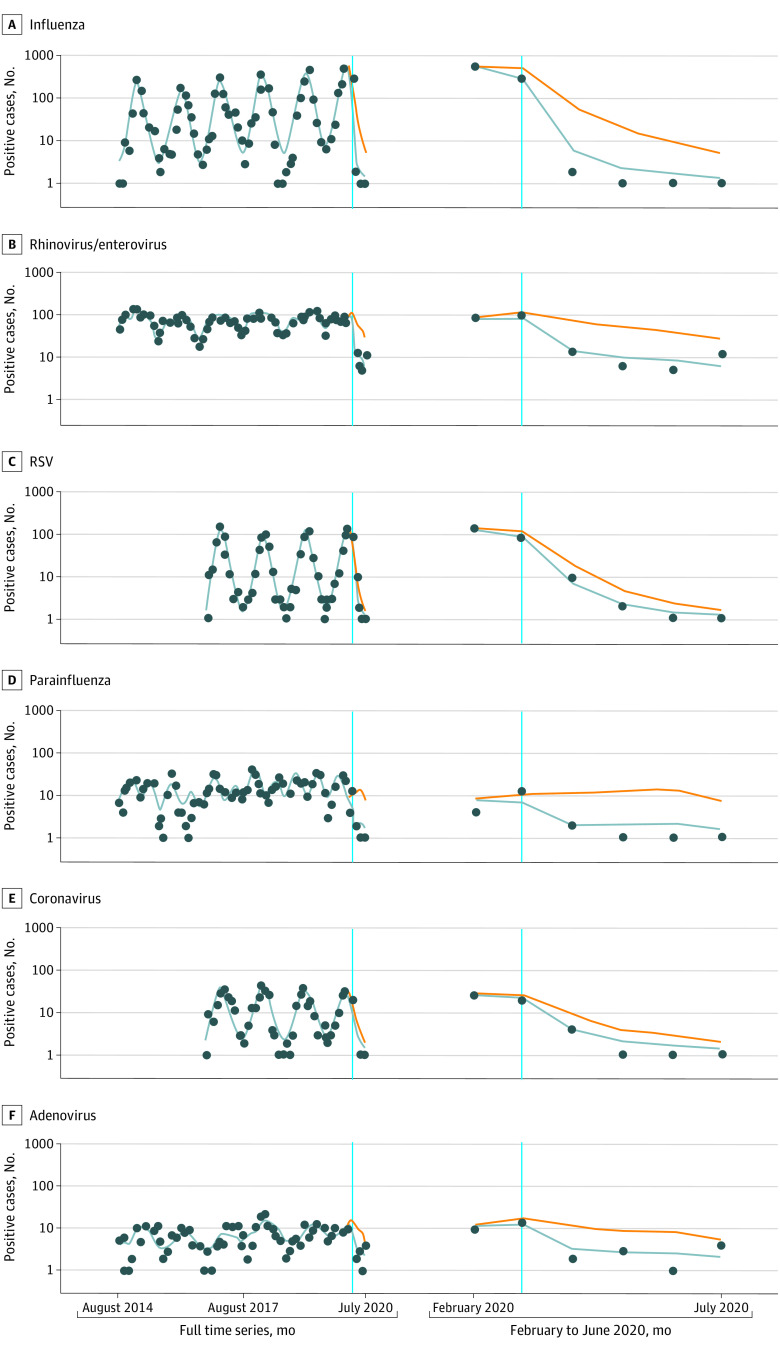
Organism-Specific Time Series Data on Observed and Projected Positive Cases in the Absence of a Shelter-in-Place Order The observed number of positive test results (blue dots), the fitted number of positive results to the seasonally adjusted model (solid blue line), and the fitted number of positive results for the counterfactual absence of a shelter-in-place order (orange line). Respiratory syncytial virus (RSV) and adenovirus data were not available for 2014 to 2016.

## Discussion

We hypothesized that the statewide shelter-in-place order that was instituted to curb the spread of SARS-CoV-2 would be associated with lower rates of common viral infections circulating in the community. In April 2020, a report by Sakamoto et al^6^ indicated that in Japan, seasonal influenza activity was lower in the 2019-2020 respiratory season compared with the 5 previous years. Studies from Taiwan, Singapore, Korea, and Thailand have also shown decreased influenza incidence after countrywide public health measures were instituted to reduce the transmission of COVID-19.^7,8,9,10^ To our knowledge, this study is the first in the United States to examine the implications of SARS-CoV-2 public health measures for influenza rates as well as the first in the world to examine the implications of these public initiatives for other seasonal respiratory viruses.

### Limitations

This study has several limitations. First, although UC Davis Health serves as a referral center for more than 6 million Northern California residents, this is a study of a single health system. Second, although the statistical analysis rigorously estimated the seasonally adjusted outcome of a shelter-in-place order as an IRR, the order was issued toward the end of the respiratory viral season in California. The implication of this public health intervention for the 2020-2021 respiratory viral season is unknown and could be altered by favorable (ie, improved awareness and acceptance of public health initiatives) or unfavorable (ie, poor adherence owing to isolation fatigue) factors. Third, health care avoidance because of concerns about COVID-19, particularly by those with milder infections, was a possible confounder in the number of laboratory-confirmed cases. Increased use of telehealth and limits on testing capabilities are other possible confounders. We used the rate of positive cases to control for fewer tests attributed to changes in health-seeking behavior. In sensitivity analyses that included daily rates of positive cases, the estimated IRRs were similar, with IRRs for parainfluenzavirus and adenovirus becoming statistically significant (eTable in the Supplement). Fourth, we were unable to remove from the analysis multiple positive polymerase chain reaction test results for the same patient; therefore, duplicate tests could falsely but slightly increase the proportion of positive test results.

## Conclusions

The restrictions on travel, mandated mask wearing, and physical distancing that are embedded in the California shelter-in-place order have been controversial because of their substantial social and economic costs.^11^ However, this cohort study shows that public health strategies have been associated with lower rates of respiratory viruses in the community served by UC Davis Health.

## Notes

Supplement. **eTable.** Seasonally Adjusted Data Following Institution of Shelter-in-Place Order With Incidence Rate of Positives Referring to Number of Cases per Day

## References

[1] Newsom G. Executive order N-33-20. Accessed March 19, 2020. https://www.gov.ca.gov/wp-content/uploads/2020/03/3.19.20-attested-EO-N-33-20-COVID-19-HEALTH-ORDER.pdf

[2] SAS Institute SAS System for Windows. 9.4 ed. SAS Institute; 2012.

[3] Barnett AG, Dobson AJ Analysing Seasonal Health Data. Springer; 2010. doi:10.1007/978-3-642-10748-1

[4] Bernal JL, Cummins S, Gasparrini A Interrupted time series regression for the evaluation of public health interventions: a tutorial. Int J Epidemiol. 2017;46(1):348-355. doi:10.1093/ije/dyw098 27283160 PMC5407170

[5] Ten Eyck P, Cavanaugh JE An alternate approach to pseudo-likelihood model selection in the generalized linear mixed modeling framework. Sankhya B. 2018;80:98-122. doi:10.1007/s13571-017-0130-5

[6] Sakamoto H, Ishikane M, Ueda P Seasonal influenza activity during the SARS-CoV-2 outbreak in Japan. JAMA. 2020;323(19):1969-1971. doi:10.1001/jama.2020.6173 32275293 PMC7149351

[7] Soo RJJ, Chiew CJ, Ma S, Pung R, Lee V Decreased influenza incidence under COVID-19 control measures, Singapore. Emerg Infect Dis. 2020;26(8):1933-1935. doi:10.3201/eid2608.201229 32339092 PMC7392467

[8] Kuo SC, Shih SM, Chien LH, Hsiung CA Collateral benefit of COVID-19 control measures on influenza activity, Taiwan. Emerg Infect Dis. 2020;26(8):1928-1930. doi:10.3201/eid2608.201192 32339091 PMC7392415

[9] Lee H, Lee H, Song KH, Impact of public health interventions on seasonal influenza activity during the SARS-CoV-2 outbreak in Korea. Clin Infect Dis. Published online May 31, 2020. doi:10.1093/cid/ciaa672 32472687 PMC7314207

[10] Suntronwong N, Thongpan I, Chuchaona W, Impact of COVID-19 public health interventions on influenza incidence in Thailand. Pathog Glob Health. 2020;114(5):225-227. doi:10.1080/20477724.2020.1777803 32521210 PMC7480427

[11] Mello MM, Greene JA, Sharfstein JM Attacks on public health officials during COVID-19. JAMA. 2020;324(8):741-742. doi:10.1001/jama.2020.14423 32777019

