# Supplementary material for: Evaluation of Seasonal Respiratory Virus Activity Before and After the Statewide COVID-19 Shelter-in-Place Order in Northern California
Source: JAMA Netw Open. 2021 Jan 25;4(1):e2035281. doi: 10.1001/jamanetworkopen.2020.35281 (PMC7835714; doi:10.1001/jamanetworkopen.2020.35281)
Supplement: Supplement. — eTable. Seasonally Adjusted Data Following Institution of Shelter-in-Place Order With Incidence Rate of Positives Referring to Number of Cases per Day [file jamanetwopen-e2035281-s001.pdf]

Supplementary Online Content

Partridge E, McCleery E, Cheema R, et al. Evaluation of seasonal respiratory virus activity before and after the statewide COVID-19 shelter-in-place order in Northern California. *JAMA Netw Open*. 2021;4(1): e2035281. doi:10.1001/jamanetworkopen.2020.35281

eTable. Seasonally Adjusted Data Following Institution of Shelter-in-Place Order With Incidence Rate of Positives Referring to Number of Cases per Day

This supplementary material has been provided by the authors to give readers additional information about their work.

©2021 Partridge E et al. *JAMA Network Open*

eTable. Seasonally Adjusted Data Following Institution of Shelter-in-Place Order With Incidence Rate of Positives Referring to Number of Cases per Day

Organism	IRR	95% CI	P-value	Percentage decrease in positive cases from expected
Influenza	0.08	0.01 – 0.64	0.02	92 (36 – 99)
Rhinovirus/Enterovirus	0.15	0.06 – 0.40	0.0003	85 (60 – 94)
RSV	0.39	0.10 – 1.55	0.18	61 (-55 – 90)
Parainfluenza	0.06	0.004 – 0.96	0.047	94 (5 – 99.6)
Coronavirus	0.37	0.06 – 2.52	0.30	63 (-152 – 94)
Adenovirus	0.20	0.04 – 0.99	0.049	80 (1 – 96)

Notes: For each organism, the IRR was estimated in a Poisson regression model for overdispersed autocorrelated monthly time series data with cosinor analysis adjustments for sinusoidal seasonal effects (12-month cycle) and additional adjustment for historical patterns. For RSV, adjustments for historical patterns were made with calendar date as a linear term and additional cosinor analysis terms for 6-month cycles were included. For all other organisms, indicator terms for each virus year (August to July) adjusted for historical patterns, with 2019-20 as reference year. For parainfluenza, additional cosinor analysis terms for 6-month cycles were added.

©2021 Partridge E et al. *JAMA Network Open*
